# Validation of an algorithm for selection of SGLT2 and DPP4 inhibitor therapies in people with type 2 diabetes across major UK ethnicity groups: a retrospective cohort study

**DOI:** 10.1016/j.lanepe.2025.101547

**Published:** 2025-11-27

**Authors:** Laura M. Güdemann, Katherine G. Young, Pedro Cardoso, Bilal A. Mateen, Rury R. Holman, Naveed Sattar, Ewan R. Pearson, Andrew T. Hattersley, Angus G. Jones, Beverley M. Shields, John M. Dennis, MASTERMIND Consortium, Lauren Rodgers, William Henley, Timothy J. McDonald, Michael N. Weedon, Nicky Britten, Catherine Angwin, Robert S. Lindsay, Christopher Jennison, Mark Walker, Kennedy Cruickshank, Salim Janmohamed, Christopher J. Hyde, Andrew Farmer, Alastair Gray, Stephen Gough, Olorunsola Agbaje, Trevelyan J. McKinley, Sebastian J. Vollmer, William Hamilton, Rhian Hopkins

**Affiliations:** a Clinical and Biomedical Sciences, University of Exeter Medical School, Exeter, UK; b School of Life Sciences, University of Birmingham, Birmingham, UK; c Diabetes Trials Unit, Radcliffe Department of Medicine, University of Oxford, Oxford, UK; d NIHR Oxford Biomedical Research Centre, Churchill Hospital, Oxford, UK; e School of Cardiovascular and Metabolic Health, University of Glasgow, Glasgow, UK; f Division of Diabetes, Endocrinology and Reproductive Medicine, Ninewells Hospital and Medical School, University of Dundee, Dundee, UK

**Keywords:** Type 2 diabetes, Precision medicine, Personalised medicine, Heterogeneous treatment effects, Ethnicity, SGLT2-inhibitors, DPP4-inhibitors

## Abstract

**Background:**

Routine clinical features of individual patients can potentially be used to guide selection of type 2 diabetes treatments. We aimed to evaluate a recently proposed treatment selection model predicting differences in glycaemic responses to SGLT2-inhibitors and DPP4-inhibitors across major UK ethnicity groups.

**Methods:**

We externally validated the SGLT2i-DPP4i model in UK primary care cohort (CPRD Aurum, 2013–2020) independent of the original model development cohort. Non-insulin treated individuals with type 2 diabetes were identified and categorised by major UK self-reported ethnicity groups: White, Black, South Asian and Mixed/Other. For each ethnicity group, we applied a closed testing procedure to assess whether model recalibration was required. After model updates, we assessed the calibration accuracy of predicted differences in glycaemic response (6-month change in HbA1c) between SGLT2i and DPP4i for each ethnicity group.

**Findings:**

SGLT2i (n = 57,749) and DPP4i (n = 87,807) initiations were identified amongst people of White (n = 114,287; 78.5%), Black (n = 6663; 4.6%), South Asian (n = 20,969; 14.4%) and Mixed/Other (n = 3637; 2.5%) ethnicities. Minor model adjustment was required to adjust for greater observed than predicted glycaemic responses to DPP4i (White—1.6 mmol/mol; Black—3.0 mmol/mol; South Asian—2.6 mmol/mol; Mixed/Other—2.6 mmol/mol). SGLT2i predictions did not require adjustment for non-White ethnicity groups. After model updates, average predicted HbA1c reduction was 3.7 mmol/mol (95% CI 3.5–3.9) greater with SGLT2i than DPP4i for those of White ethnicity; this was greater than for those of South Asian (2.1 mmol/mol (95% CI 1.6–2.6)), Black (0.6 mmol/mol (95% CI 0.5–1.7)) and Mixed/Other (2.6 mmol/mol (95% CI 1.4–3.8)) ethnicity groups. For all ethnicity groups, predicted differential glycaemic treatment effects were well calibrated.

**Interpretation:**

Our model for selection of SGLT2-inhibitor and DPP4-inhibitor therapies was accurate for all major self-reported ethnicity groups in a UK primary care cohort. Simple recalibration is beneficial to optimise performance and this is recommended prior to deployment of the model in new populations and settings.

**Funding:**

10.13039/501100000265UK Medical Research Council, National Institute for Health and Care Research Exeter Biomedical Research Centre, and 10.13039/501100001648EFSD/Novo Nordisk.

**Research in context**

**Evidence before this study**

In type 2 diabetes, multiple glucose-lowering treatments are available and generalisable precision medicine approaches to optimise choice of treatment for individual patients are needed. We recently developed and externally validated a treatment selection algorithm for Sodium-Glucose Cotransporter-2 inhibitors (SGLT2i) and Dipeptidyl peptidase-4 inhibitor (DPP4i) therapies to predict which of the two treatments is optimal for glucose-lowering for individual patients based on five routinely measured clinical features (baseline HbA1c, age, BMI, eGFR, ALT). Although validation suggested clear potential to support decisions on optimal treatment in population-based and clinical trial datasets, a limitation is that the model was developed in a predominantly White population and performance across different races or ethnicities was not assessed. For the algorithm to provide an equitable approach to T2D treatment, evaluation of accuracy across diverse populations is a required next step. Other evidence on variation in glucose-lowering response for SGLT2i and DPP4i by race or ethnicity is sparse. We searched PubMed/Medline for articles of all languages published between 1 January 2004 and 29 October 2025 using the terms ((Diabetes Mellitus[MeSH Major Topic] AND (“Precision” OR “stratification” OR “personalis∗” OR “targeted” OR “effect heterogeneity” OR “individualis∗” OR “heterogenous treatment effects” OR “treatment selection” OR “differential response” OR “treatment effect∗” OR “glucose-lowering efficacy” OR “glucose lowering effect∗” OR “glycemic” OR "HbA1c") AND ((Sodium-Glucose Transporter 2 Inhibitors[MeSH Major Topic] OR SGLT-2 or SGLT2) OR (Dipeptidyl-Peptidase IV Inhibitors[MeSH Major Topic]) OR DPP-4 OR DPP4) AND ((Ethnic Groups[MeSH Terms] OR ethnic∗ OR race OR racial OR ethnicity OR Caucasian OR White OR Black OR African OR Asian OR Hispanic OR Latino OR “mixed ethnic∗” OR “minority groups”)))). We found no studies developing or validating models to predict HbA1c outcomes with SGLT2i and DPP4i in different ethnicity groups. Two meta-analysis studies reported that glycaemic response to DPP4i is higher in people of Asian versus white ethnicity, and two meta-analysis studies reported that glycaemic response to SGLT2i may be similar in people of White, Asian and Black ethnicities.

**Added value of this study**

We have assessed the accuracy of the SGLT2i and DPP4i treatment selection algorithm across major UK ethnicity groups (White [n = 1,142,887], South Asian [n = 20,969], Black [n = 6663], and Mixed/Other [n = 3637]) in an external validation study. We used a large UK population-based cohort of people with T2D and near complete capture of self-reported ethnicity. We developed a generalisable procedure to test if recalibration of the original algorithm was required for each ethnicity group. This showed that the algorithm required modest adjustment to account for a 2–3 mmol/mol greater glucose-lowering response to DPP4i (6-month HbA1c change from baseline) in South Asian, Black, and Mixed/Other groups compared to the response predicted by the original algorithm. After model adjustment, algorithm predictions of differences in HbA1c response between SGLT2i and DPP4i were accurate for all the ethnicity groups, with clinically relevant differences in predicted response to the two therapies that could support individualised targeting of treatment.

**Implications of all the available evidence**

Findings support the generalisability of treatment selection models based on simple routine clinical features across diverse populations of people with T2D. Better informing choice of T2D therapy based on optimising glucose-lowering response and using routine clinical features represent an equitable and low-cost precision medicine approach with potential worldwide applicability.

## Introduction

Individualised treatment recommendations for intensifying glucose-lowering therapy to improve glycaemic control in people with type 2 diabetes (T2D) are currently lacking.^1^ There are a number of different treatment options recommended after first-line metformin, but little guidance to aid prescribing decisions for the majority of patients. Treatment decisions are therefore challenging and therapeutic inertia is a major problem.^2^ A precision medicine approach, which utilises individual characteristics, can help tailor treatment selection to target those most likely to benefit. This strategy aims to optimise outcomes by personalising care based on specific patient profiles. Dipeptidyl peptidase-4 inhibitor (DPP4i) and Sodium-Glucose Cotransporter-2 inhibitors (SGLT2i) are recommended for treatment intensification after metformin^3^ and represent around 60% of treatment initiations with a second-line treatment in the UK.^4^ Hence, their direct comparison in prediction models is clinically relevant.

In a recent study, we developed and validated a novel treatment selection algorithm for SGLT2i and DPP4i therapies. This treatment selection model provides individualised estimates of relative glucose-lowering effect (6-month HbA1c response) based on five routinely measured clinical features (baseline HbA1c, current age, body-mass-index (BMI), estimated glomerular filtration rate (eGFR), and alanine aminotransferase (ALT)).^5^ It was developed in a large UK population-based cohort and validated in head-to-head randomised clinical trials.^5^ Validation showed that the proposed treatment selection model can identify a subgroup of around 40% of people of the study cohort who experience a glycaemic benefit of 5 mmol/mol or greater on SGLT2i compared with DPP4i. A smaller group of around 15% of patients have a greater glycaemic benefit and a lower risk of short-term discontinuation with DPP4i.

A limitation of the algorithm was that model performance was not evaluated across people of different ethnicities due to the relatively low numbers of individuals of non-White ethnicities in the original study datasets.^5^ It is therefore important to assess the validity of the proposed treatment selection model across different ethnicity groups to lower risk of algorithmic bias and potential suboptimal or inequitable treatment recommendations for minority populations.^6^, ^7^, ^8^ Evidence from previous studies investigating possible treatment effect heterogeneity for different ethnicity groups is sparse but, some studies suggest that, whilst glycaemic reductions with the SGLT2i class may not vary by ethnicity,^7^^,^^9^, ^10^, ^11^, ^12^ DPP4i glycaemic reductions may be greater in people of Asian ethnicity compared with people of White ethnicity^13^ or non-Asian patient subgroups.^14^ We, therefore, aimed to perform an external validation study to assess and optimise the performance of the SGLT2i and DPP4i therapy treatment selection algorithm across major UK ethnicity groups.

## Methods

### Research design and participants

Routine clinical data from 960 UK primary care practices from 1 January 2013 to 6 November 2020 were accessed from Clinical Practice Research Datalink (CPRD) Aurum. These data comprised a near distinct set of individuals to our development set (CPRD Gold), as the two datasets were collected from different GP practice electronic patient record software systems.^15^ Study eligible patients were identified using the same inclusion criteria as previously described: individuals with type 2 diabetes, not insulin-treated, eGFR >45, baseline HbA1c >53 and <120 mmol/mol.^5^ Further details on cohort construction and eligibility criteria are given in Supplementary Figure S1, with the underlying code available at https://github.com/Exeter-Diabetes/CPRD-LauraJohn-SGLT2iDPPi-ethnicity.

Ethnicity was defined using self-reported data from primary care, supplemented with linked hospital admissions data where missing (recommended by Mathur et al.^16^). Patients were categorised into the major UK ethnicity groups: White, South Asian, Black, and Mixed/Other (combined due to low numbers).^17^ For individuals with codes for multiple recorded ethnicities, we used the group with the highest number of recorded codes; if counts were equal we used the most recent code. Further details on this coding strategy can be found here: https://github.com/Exeter-Diabetes/CPRD-Codelists/blob/main/readme.md#ethnicity.

### Outcomes

The outcomes matched those of the original model development study.^5^ The primary outcome comprised achieved 6-month HbA1c defined as the closest HbA1c measure to 6 months of treatment initiation within a window of 3–15 months, on stable therapy (no change in co-therapy in the 90 days prior to initiating the treatment of interest, no addition or cessation of other glucose-lowering medication and continued prescription of the drug of interest).

Secondary outcomes included 6-month weight change and treatment discontinuation within 6-months of initiation (as a proxy for non-tolerability). Each outcome was analysed using an outcome-specific cohort with complete cases for the outcome and all relevant baseline characteristics, as explained in Supplementary Figure S1.

### Model predictors and other clinical features

A detailed description of the model as originally developed can be found in the Supplementary material.^5^ Extracted clinical features at drug initiation comprised: age, sex, diabetes duration, Index of Multiple Deprivation (IMD) quintile,^18^ baseline HbA1c, BMI, eGFR, ALT, the number of current, and ever, prescribed glucose-lowering drug classes. We required complete data for all predictor variables, informed by recent research showing comparable model performance with missing data imputation.^19^

### Statistical analysis

We implemented a 3-step approach for ethnicity-specific model optimisation and performance assessment.

#### Step 1: determining the need for model updating in each ethnicity group and drug class

We first evaluated the accuracy of predicted 6-month HbA1c outcomes from the original model^5^ versus observed 6-month HbA1c outcomes for each drug class, separately for each ethnicity group. The requirement for model updates to improve prediction of 6-month HbA1c outcome by ethnicity was assessed using a closed testing procedure informed by Vergouwe et al.^20^ We employed a sequence of log-likelihood tests to determine whether the original treatment selection model required recalibration.

The original model (Model 1) was compared with two recalibrated models: recalibration-in-the-large (Model 2, updated model intercept) and intercept/slope recalibration (Model 3, updated intercept and overall calibration slope). For Model 2, updated intercepts for each drug class were calculated by fitting an intercept-only linear regression model, with the difference between observed HbA1c and predicted HbA1c from the original model as the outcome. The estimated intercept from this model represented the average difference between the observed and predicted HbA1c outcomes. Model 3 recalibration was performed by regressing the observed HbA1c outcome against the predicted HbA1c outcome from the original model.

To test whether the updated ethnicity-specific recalibrated models led to improvements over the original model, three model comparisons were performed using a nominal p-value for α = 5%.^20^ The first test compared the prediction performance of Model 1 (original model) versus the Model 2 (recalibration-in-the-large model). The Model 2 versus Model 1 test was performed using a log-likelihood test with m degrees of freedom, where m is the number of estimated parameters in the original model (intercept not considered). The second test compared Model 2 with Model 3 (updated intercept and slope), and the test was performed with m + 1 degrees of freedom.^20^

We then applied the following decision rules to perform ethnicity-specific updates for predictions for each drug class:

• if the second test was significant, Model 3 (updated intercept and slope) was chosen,
• if the first but not the second test was significant, Model 2 (updated intercept) was chosen,
• if none of the tests were significant, Model 1 (original model) was chosen.

Further details on the sequential testing procedure can be found in the Supplementary material.

#### Step 2: assessment of calibration accuracy of predicted differences by drug class in 6-month HbA1c

For each ethnicity group, model calibration accuracy for predicting differences between the two drug classes in 6-month HbA1c was assessed by sub-dividing cohorts into deciles defined by predicted HbA1c differences, and then, within each decile, comparing mean predicted HbA1c differences to mean HbA1c differences between individuals receiving SGLT2i and DPP4i in practice. This validation approach followed the original study^5^ and was required as counterfactual outcomes for each therapy in individual patients were not available.

Firstly, we used the model to predict HbA1c outcome on both treatments for each individual in the study cohort. Secondly, we calculated individual-level predicted HbA1c differences as the difference between SGLT2i and DPP4i model predictions for each patient. Thirdly, we stratified individuals within each ethnicity group based on deciles of predicted HbA1c difference. Finally, we estimated average HbA1c differences between individuals receiving SGLT2i and DPP4i within decile group, with adjustments to account for the fact that the individuals receiving these two different drug classes were not matched. These average HbA1c differences were estimated from a linear regression model with achieved HbA1c as the outcome, received drug class as the main effect, and adjusted for baseline HbA1c, number of other current glucose-lowering drugs, number of glucose-lowering drug classes ever prescribed, month outcome HbA1c measurement was taken, eGFR, log of ALT, current age and baseline BMI (continuous features modelled as 3-knot restricted cubic splines).

To evaluate model performance for predicting absolute (rather than differential) HbA1c outcomes, we plotted predicted versus observed HbA1c outcomes and estimated root mean squared errors to represent the average difference between predicted and observed values.

#### Step 3: estimation of unadjusted differences in 6-month HbA1c response (change from baseline in HbA1c), weight change and early discontinuation

Individuals in the study cohort were stratified into five subgroups defined using clinically relevant thresholds of predicted individual glycaemic benefit (benefit with SGLT2i versus DPP4i: ≥5 mmol/mol, 3–5 mmol/mol, 0–3 mmol/mol; benefit with DPP4i versus SGLT2i: 0–3 mmol/mol, ≥3 mmol/mol). For each subgroup, we estimated unadjusted mean glycaemic response, weight change, and discontinuation proportions by drug class.

### Sensitivity analysis

As Mixed and Other ethnicity groups were pooled together for the main analysis due to relatively low numbers, we explored calibration accuracy of both groups separately in sensitivity analysis. Although deprivation was not included in the original model and therefore our primary validation, we were able to assess the impact of deprivation on ethnicity specific model performance by additionally including IMD quintile as a covariate when estimating average HbA1c differences. Finally, we additionally assessed model performance when evaluating longer-term 12-month HbA1c outcomes (closest HbA1c record to 12 months within 3–15 months of treatment initiation).

All analyses were conducted using R (version 4.4.1), and all analysis code can be found online at: https://github.com/Exeter-Diabetes/CPRD-LauraJohn-SGLT2iDPPi-ethnicity. We followed TRIPOD prediction model reporting guidance.^21^

### Ethics statement

Clinical Practice Research Datalink was obtained under licence from the UK Medicines and Healthcare products Regulatory Agency. CPRD data is provided by patients and collected by the NHS as part of their care and support. Approval for CPRD data access and the study protocol was granted by the CPRD Independent Scientific Advisory Committee (eRAP protocol number: 24_004747).

### Role of the funding source

The funder of the study had no role in study design, data collection, data analysis, data interpretation, or writing of the report.

## Results

The study cohort included 145,556 drug initiations of SGLT2i (n = 57,749) and DPP4i (n = 87,807) (patient flowchart: Supplementary Figure S1). 78.5% (n = 114,287) were of White, 4.6% (n = 6663) of Black, 14.4% (n = 20,969) of South Asian and 2.5% (n = 3637) of Mixed/Other ethnicity. Baseline characteristics for the HbA1c outcome cohort are shown in Table 1.

**Table 1:** Baseline clinical characteristics of the study cohort for treatment selection model validation of HbA1c outcome.

	DPP4i (N = 61,440)	SGLT2i (N = 36,047)
Current age, years	62.8 (12.0)	58.5 (10.4)
Duration of diabetes, years	8.9 (6.7)	9.2 (6.3)
Sex		
Female	23,652 (38.5%)	13,578 (37.7%)
Male	37,788 (61.5%)	22,469 (62.3%)
Ethnicity		
White	48,832 (79.5%)	28,497 (79.1%)
South Asian	8397 (13.7%)	5209 (14.5%)
Black	2780 (4.5%)	1409 (3.9%)
Mixed or Other	1431 (2.3%)	932 (2.6%)
Index of multiple deprivation quintile		
1 (least deprived)	10,295 (16.8%)	6102 (16.9%)
2	11,149 (18.1%)	6439 (17.9%)
3	11,760 (19.1%)	6956 (19.3%)
4	13,710 (22.3%)	8031 (22.3%)
5 (most deprived)	14,493 (23.6%)	8502 (23.6%)
Not available	33 (0.05%)	17 (0.05%)
DPP4-inhibitor type		
Alogliptin	12,923 (21.0%)	–
Linagliptin	12,926 (21.0%)	–
Saxagliptin	3686 (6.0%)	–
Sitagliptin	31,388 (51.1%)	–
Vildagliptin	517 (0.8%)	–
SGLT2-inhibitor type		
Canagliflozin	–	6489 (18.0%)
Dapagliflozin	–	15,642 (43.4%)
Empagliflozin	–	13,891 (38.5%)
Ertugliflozin	–	25 (0.1%)
Number of glucose-lowering drug classes ever prescribed		
3	27,638 (45.0%)	8610 (23.9%)
3	25,993 (42.3%)	11,387 (31.6%)
4+	7809 (12.7%)	16,050 (44.5%)
Number of other current glucose-lowering drugs		
0	3473 (5.7%)	1182 (3.3%)
1	36,017 (58.6%)	14,578 (40.4%)
2	21,228 (34.6%)	16,286 (45.2%)
3	722 (1.2%)	4001 (11.1%)
Background therapy		
Metformin	55,087 (89.7%)	32,995 (91.5%)
Sulphonylurea	22,267 (36.2%)	12,959 (36.0%)
DPP4-inhibitor	–	10,438 (29.0%)
SGLT2-inhibitor	1687 (2.7%)	–
Thiazolidinedione	1264 (2.1%)	910 (2.5%)
GLP-1 receptor agonist	201 (0.3%)	1847 (5.1%)
Baseline biomarkers		
HbA1c, mmol/mol	72.2 (13.4)	76.0 (14.3)
BMI, kg/m^2^	31.8 (6.4)	33.7 (6.7)
eGFR, mL/min per 1.73 m^2^	88.6 (17.8)	95.1 (14.5)
Alanine transaminase, IU/L	3.3 (0.5)	3.4 (0.5)
HbA1c outcome		
HbA1c, mmol/mol	63.6 (15.5)	64.2 (14.1)
Month of HbA1c measure	6.7 (2.8)	6.6 (2.7)

Baseline clinical characteristics for the secondary outcome cohorts are shown in Supplementary Tables S1 and S2. Data are mean (1 SD) for continuous variables.

### A model update was required to adjust for a greater glycaemic response to DPP4i in people of non-white ethnicity

The model updating procedure identified greater observed average glycaemic response with DPP4i than predicted by the original model, which was most pronounced in individuals of non-White ethnicity. Model intercept recalibration (Model 2) was therefore performed to allow for greater DPP4i response for all ethnicity groups, as follows: White −1.6 mmol/mol; Black −3.0 mmol/mol; South Asian −2.6 mmol/mol; Mixed/Other −2.6 mmol/mol. For individuals initiating SGLT2i across non-White ethnicity groups, the original model fitted well, and no model updates were required. For the White ethnicity group initiating SGLT2i, a −0.9 mmol/mol intercept adjustment was applied. A summary of the full testing procedure results is provided in Supplementary Table S3.

### Model predicted HbA1c differences are accurate for all ethnicity groups

**Fig. 1:**
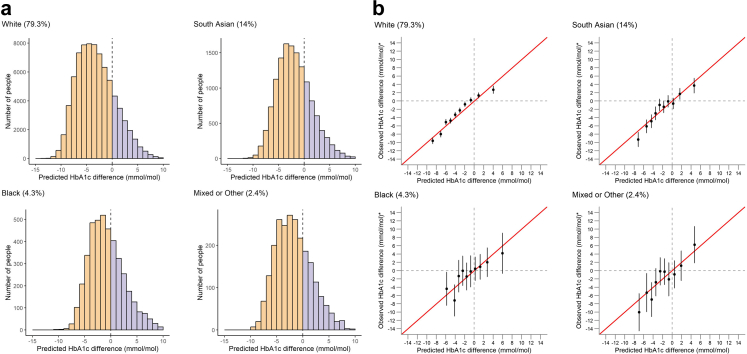
SGLT2i-DPP4i treatment selection model performance in CPRD clinical data. Negative values reflect a predicted 6-month HbA1c benefit on SGLT2-inhibitor treatment, positive values reflect a predicted 6-month HbA1c on DPP4-inhibitor treatment. (a) Distribution of predicted individual-level differential treatment effects for 6-month HbA1c of SGLT2i-inhibitor treatment compared to DPP4-inhibitor treatment, by UK ethnicity group. (b) Calibration between observed and predicted 6-month HbA1c treatment effects by decile of predicted treatment effect, by UK ethnicity group. Red lines represent perfect calibration. Point estimates represent average HbA1c differences for subgroups defined by decile of predicted treatment benefit, with average HbA1c differences estimated as adjusted absolute mean differences in 6-month HbA1c outcome between individuals receiving each drug class. Bars represent 95% confidence intervals.

Fig. 1a shows the distribution of the differential treatment effects predicted from the updated treatment selection model. In all ethnicity groups, a majority of individuals were predicted to have a higher glycaemic benefit on SGLT2i compared to DPP4i (63%–79% across ethnicity groups). The average predicted response was 3.7 mmol/mol (95% CI 3.5–3.9) greater with SGLT2i than DPP4i for those of White ethnicity; this was greater than for those of South Asian (2.1 mmol/mol (95% CI 1.6–2.6)), Black (0.6 mmol/mol (95% CI 0.5–1.7)) and Mixed/Other (2.6 mmol/mol (95% CI 1.4–3.8)) ethnicity groups.

The results of the calibration assessment for the updated model are shown in Fig. 1b. Within deciles of predicted individualised treatment effect, adjusted average HbA1c differences and predictions from the updated model were generally similar across all ethnicity groups, indicating that the predicted individualised treatment effects were well calibrated. However, minor miscalibration was observed in the South Asian ethnicity group, with findings suggestive of a greater HbA1c benefit with SGLT2i than predicted in the decile with the highest predicted HbA1c benefit with SGLT2i over DPP4i in Fig. 1b.

For predicting direct HbA1c outcome rather than differences in HbA1c outcome by drug class, root mean squared errors were 12.5 mmol/mol for the White, 12.3 mmol/mol for South Asian, 15.5 mmol/mol for Black and 12.7 mmol/mol for the Mixed/Other ethnicity groups, with good average calibration between observed and predicted absolute HbA1c outcome (Supplementary Figure S2).

### Across all ethnicity groups, the model identifies clinically meaningful differences in HbA1c response, including a subgroup with negligible response to SGLT2i

**Fig. 2:**
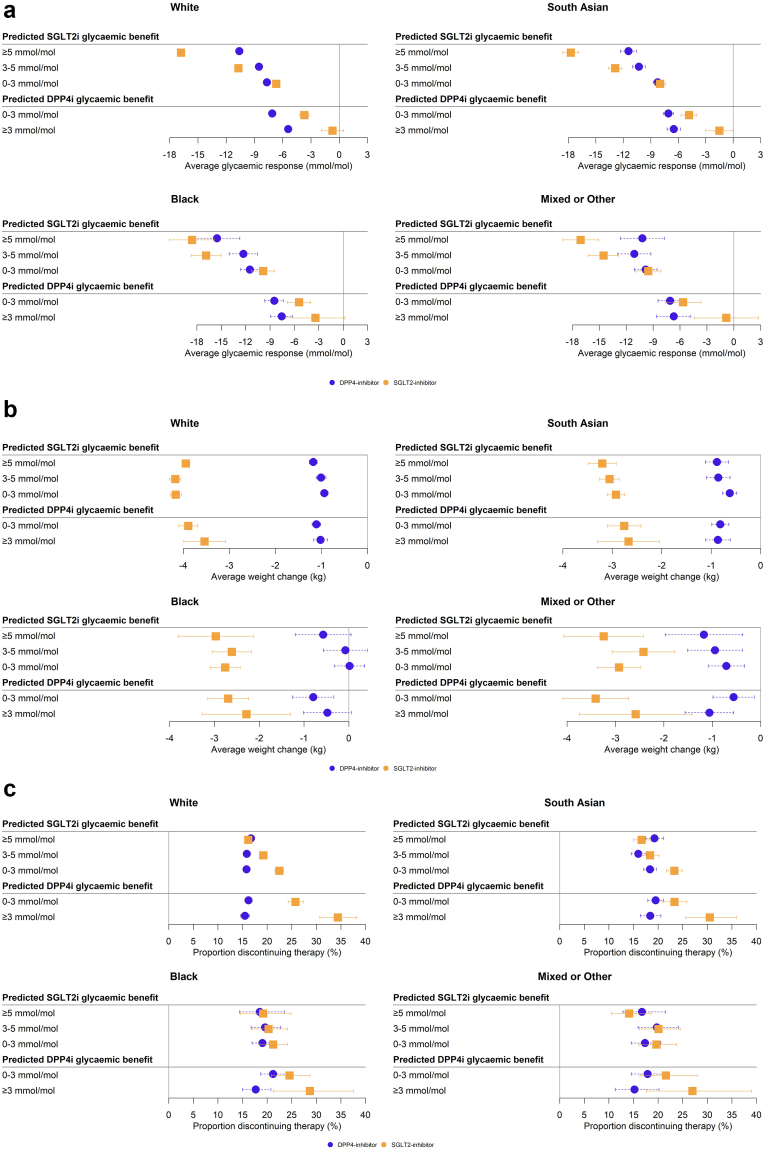
6-month HbA1c response, weight change and risk of treatment discontinuation across subgroups defined by clinical cutoffs of predicted treatment benefit, by UK ethnicity group, in CPRD clinical data. (a) HbA1c response (mmol/mol), estimated as unadjusted mean change from baseline in 6-month HbA1c. (b) Weight change (kg), defined as unadjusted mean change from baseline in 6-month weight. (c) Treatment discontinuation (%), defined as the unadjusted proportion of individuals discontinuing treatment within 6 months. Bars represent 95% confidence intervals. Supplementary Tables S4–S6 report the underlying results for HbA1c response, weight change, and treatment discontinuation.

Fig. 2a shows observed mean 6-month HbA1c response to each drug class in subgroups defined by predicted differential glycaemic response for each ethnicity group. Differences in predicted response between the two drug classes were broadly consistent with observed differences. Subgroups predicted to have a benefit on SGLT2i greater than 5 mmol/mol actually achieved a HbA1c benefit on SGLT2i of 6.2 mmol/mol (95% CI 5.8–10.4) higher compared to on DPP4i in the White ethnicity group, 3.1 mmol/mol (95% CI 1.0–12.9) in the Black ethnicity group, 6.3 mmol/mol (95% CI 5.1–12.9) in the South Asian ethnicity group, and 6.9 mmol/mol (95% CI 3.7–8.0) in the Mixed/Other ethnicity group. Individuals predicted to have a benefit on DPP4i of more than 3 mmol/mol achieved an average glycaemic benefit of 4.7 mmol/mol (95% CI 3.4–6.0) in the White ethnicity group, 4.2 mmol/mol (95% CI 0.4–8.0) in the Black ethnicity group, 5.0 mmol/mol (95% CI 3.2–6.8) in the South Asian ethnicity group, and 5.9 mmol/mol (95% CI 1.3–10.4) in the Mixed/Other ethnicity group higher than on SGLT2i.

Interestingly, in those predicted to have a DPP4i benefit of >3 mmol/mol, this appeared to be driven by a lack of meaningful response to SGLT2is in this subgroup (−0.7 mmol/mol (95% CI −1.9 to 0.5) for White ethnicity, −3.4 mmol/mol (95% CI −7.0 to 0.2) for Black ethnicity, −1.5 mmol/mol (95% CI −3.0 to 0.0) for South Asian ethnicity and −0.8 mmol/mol (95% CI −4.4 to 2.8) for Mixed/Other ethnicity). This subgroup represented 6.6% of those of White, 8.5% of South Asians, 13.4% of Black, and 9.5% of Mixed/Other ethnicities (Supplementary Table S4).

### All ethnicity groups have a greater weight loss with SGLT2i versus DPP4i, but those with the greatest predicted glycaemic benefit with DPP4i have a higher rate of discontinuation if initiating SGLT2i

Fig. 2b shows consistently higher weight reduction with SGLT2i versus DPP4i across subgroups defined by predicted differential glycaemic response for all ethnicity groups. Similarly, differences in patterns of early discontinuation were similar by ethnicity, with higher discontinuation for people initiating SGLT2i in those with a predicted benefit on DPP4i, although this was most pronounced for individuals of White ethnicity group (Fig. 2c).

### Sensitivity analysis

Calibration accuracy of predicted HbA1c differences were similar when Mixed and Other ethnicity groups were analysed separately (Supplementary Figure S3), and for each of the main ethnicity groups when adjusting for deprivation (Supplementary Figure S4), and when evaluating 12-month HbA1c outcome (Supplementary Figure S5).

## Discussion

In this external validation study, we show that a model designed to determine the optimal choice of SGLT2i and DPP4i therapies to maximise glycaemic response in people with type 2 diabetes is accurate across major UK ethnicity groups. Modest recalibration was required to reflect a greater average glycaemic response with DPP4i than predicted by the original model, of around 2–3 mmol/mol for South Asian, Black, and Mixed/Other ethnicity groups. No model updates were required for SGLT2i initiations across all non-White ethnicity groups. Importantly, good performance was achieved without adjustment of the effect of individual model predictors themselves, suggesting that clinical factors altering the response to both therapies are consistent across ethnicity groups. The application of the updated model identified an interesting patient subgroup, representing 6–13% of individuals across the ethnicity groups, with a significant HbA1c response to DPP4i but only a very limited response to SGLT2i inhibitors. Weight change and short-term discontinuation patterns were consistent across ethnicities, with greater weight loss associated with SGLT2i and higher rates of discontinuation in individuals initiating SGLT2i who were predicted to derive a greater glycaemic benefit from DPP4i. Overall, findings support the potential clinical deployment of the SGLT2i–DPP4i model for targeting treatment in diverse populations. Our methodology also provides an example of a principled approach for the external validation of treatment selection model to support translational precision medicine.

There is limited evidence from previous studies about differential glycaemic reductions with SGLT2i versus DPP4i across ethnicity groups. Previous trial meta-analysis report DPP4i to have a greater glycaemic efficacy in people of Asian ethnicity than in other ethnicity groups,^14^^,^^22^ as we found in this study, suggesting that variation in response may reflect differences in underlying pathophysiology. Our sensitivity analysis adjusting for deprivation further supports the interpretation that differences are more likely to reflect pathophysiological differences than health inequalities. Potential mechanisms include a mediation of DPP4i efficacy by clinical factors associated with insulin sensitivity, such as differences between ethnicity groups in BMI, β-cell function (HOMA-%B), or the amount of visceral fat. A further suggestion is that there may be differences in the pharmacokinetic properties of DPP4i in Asian and non-Asian groups due to variations in body size.^14^^,^^22^ Despite this enhanced DPP4i response, our results show that SGLT2i still have a greater glycaemic benefit compared to DPP4i for most people across all ethnicity groups. Similar to previous studies, our results did not show clear treatment effect heterogeneity with SGLT2i by ethnicity group.^9^, ^10^, ^11^, ^12^^,^^23^ Overall, the DPP4i-specific ethnicity differences identified in this study, although relatively modest on average, highlight the importance of future research to identify differential T2D drug effects by ethnicity and to interrogate potential underlying mechanisms.

Considering the secondary outcomes we assessed, SGLT2i have been consistently shown to lead to greater weight loss compared with DPP4i across different populations.^24^^,^^25^ This finding was consistent in our study across all ethnicity groups, including when stratified by glycaemic benefit. Whilst limited previous studies have shown lower rates of discontinuation with SGLT2i versus DPP4i,^26^^,^^27^ they did not analyse differential discontinuation risk by ethnicity. In this study, we focused on overall treatment discontinuation as a proxy for tolerability, which can be evaluated consistently across both therapies to enable a fair head-to-head comparison. Assessing heterogeneity in risk of drug-specific side effects, such as genital infections or osmotic symptoms with SGLT2i, was beyond the scope of our analysis but represents an important direction for future research, ideally using causal inference methods.^28^

A major strength of our study is the use of a large and diverse population-based cohort external to that used for model development, which included near complete data on self-reported ethnicity, and incorporation of a substantial number of individuals from non-White ethnic groups allowing for ethnicity subgroup level assessment of differential treatment effects. Recent analyses by the UK Office for National Statistics have highlighted ecosystem-wide challenges in the accuracy of routinely collected ethnicity data,^29^ and thus, it is important to recognise that completeness alone is an imperfect metric for quality in this context. No linkage to the 2022 ONS census ethnicity data currently exists for CPRD (a potential limitation of this study, as this would be considered the gold standard record for self-reported ethnicity). For when these linkages become available, we have provided a robust, fully-documented, open-source pipeline for processing and analysing electronic health record data, to support reproducibility,^30^ and replication of our results.

Another strength of this study is the application of a robust testing procedure to assess model update requirements.^20^ This procedure makes it possible to adapt prediction models for treatment selection using a data-driven approach, allowing performance optimisation in study populations that differ from the population in which the model was initially developed. Importantly, the procedure allowed us to flexibly update the model, allowing multiple potential updating strategies to be fairly evaluated. An important limitation of our study is that it was not possible to validate individual differential treatment effects due to a lack of counterfactual outcome measurements, meaning that our validation is based on subgroup-level averages rather than individual patient-level differences. This observational study also leaves the possibility for residual bias in particular relating to unmeasured prescription biases. Additionally, it was not possible to account for drug adherence or dose variation as the granularity of recorded dose data in our dataset did not allow for reliable ascertainment of medication dose or coverage. Based on previous RCT results we would expect the dose effect on HbA1c response to be relatively modest, and our previous work has demonstrated good overall model performance in multinational trial data with participants randomised to therapy and protocol driven follow-up.^5^

A further limitation is the less precise effect estimation evident in the smaller Black and Mixed/Other ethnicity groups, and our pragmatic choice of pooling Mixed and Other ethnicity groups due to low numbers. Although our analysis suggests that this choice is reasonable and the model calibrates well in all groups, larger and more diverse datasets would be of benefit for further model validation across other populations. We also did not evaluate heterogeneity in cardiorenal outcomes, although our methods could be extended to these longer-term outcomes in future work to allow a more holistic consideration of treatments that may best benefit individual patients. A final limitation is our focus on validation of the two-drug SGLT2i and DPP4i treatment model, considering we recently published a five-drug class model also including GLP-1 receptor agonists, sulfonylureas, and thiazolidinediones.^31^ The current study provides the methodological framework to perform future validation of the five-drug class model both by ethnicity in the UK when sufficient additional longitudinal data are available, and in other populations and settings beyond the UK.

Results of this study suggest that individualised treatment selection models relying on simple routine clinical features may have utility as equitable low-cost approaches for informing T2D treatment decisions in diverse populations.^31^ T2D management requires multifactorial decision making with respect to HbA1c targets, drug-specific side effects, and cardiovascular and renal health outcomes. The strength of our model therefore lies in providing additional novel information on personalised glucose-lowering which can be made available alongside evidence on drug-specific side-effect profiles, cardiovascular, and kidney benefits. To support clinical deployment, setting and population-specific model validation following the methodological framework we provide in this study will help ensure model accuracy for informing clinical decisions, and a prospective effectiveness trial would ideally be performed. Furthermore, future efforts need to focus on developing easy to use and ideally no-cost software which is required for widespread and equitable deployment of models in clinical practice.

### Conclusion

A SGLT2i–DPP4i treatment selection model accurately predicts differences in HbA1c responses in all major UK ethnicity groups. Model evaluation shows that in all ethnicity groups, a majority of individuals were predicted to have a greater glycaemic reduction on SGLT2i compared with DPP4i. The benefit of SGLT2i is however less marked in non-White ethnicity groups due to a greater response to DPP4i. Individualised treatment selection models have potential to provide a low-cost, pragmatic approach to T2D precision medicine with applicability across diverse populations.

## Contributors

LMG, JMD, BS and AGJ designed the study and developed the analysis strategy. LMG and JMD conducted the analysis. PC supported the application of the testing procedure and KGY prepared the CPRD data and constructed the T2D cohort. LMG drafted the original version of the paper which all authors helped to edit. BAM, RRH and NS provided critical revisions of the manuscript. ERP and ATH provided valuable clinical insights and helped interpret the results. LMG and JMD had access to the data and verified the data. LMD and JMD were responsible for the decision to submit the manuscript. All authors participated in editing and revising the manuscript draft and have read and approved the final version. LMG and JMD are the guarantors of this work and as such, had full access to all the data in the study and take responsibility for the integrity of the data and the accuracy of the data analysis.

## Data sharing statement

CPRD Aurum data are available by application to the CPRD Independent Scientific Advisory Committee. R code to preproduce the analysis in this paper is available at https://github.com/Exeter-Diabetes/CPRD-LauraJohn-SGLT2iDPPi-ethnicity.

## Declaration of interests

BAM holds an honorary professorial appointment at the University of Birmingham and is an employee of PATH. BAM declares grants from the UK Medical Research Council, Health Data Research UK, British Heart Foundation, the UK Engineering and Physical Sciences Research Council, the Gates Foundation, USAID, US CDC, GIZ, Wellcome Trust, Rockefeller Foundation, The Sall Family Foundation, and FCDO. RRH reports personal fees from Lilly, Merck KGaA, MitoRx, Novartis and Owen Mumford Ltd. NS declares personal fees from Abbott Diagnostics, Afimmune, Amgen, AstraZeneca, Boehringer Ingelheim, Eli Lilly, Hanmi Pharmaceuticals, MSD, Novartis, Novo Nordisk, Pfizer, and Sanofi; and grants from AstraZeneca, Boehringer Ingelheim, Novartis, and Roche Diagnostics. NS has consulted for and/or received speaker honoraria from Abbott Laboratories, AbbVie, Amgen, AstraZeneca, Boehringer Ingelheim, Carmot Therapeutics, Eli Lilly, GlaxoSmithKline, Hanmi Pharmaceuticals, Menarini-Ricerche, Metsera, Novartis, Novo Nordisk, Pfizer, and Roche; and received grant support paid to his University from AstraZeneca, Boehringer Ingelheim, Novartis, and Roche outside the submitted work. ERP reports personal fees from Lilly and Novo Nordisk. AGJ was supported by an NIHR Clinician Scientist fellowship (CS-2015-15-018) and declares research funding from the UK Medical Research Council, Diabetes UK (charity), Juvenile Diabetes Research Foundation (charity), the European Foundation for the Study of Diabetes (charity) and the Novo Nordisk Foundation (Denmark), UK National Institute of Health and Care Research, and Breakthrough Type 1 diabetes. AGJ is also an advisory board member at Novo Nordisk Foundation UK. BMS declares research funding from the UK Medical Research Council, UK National Institute for Health Research, Diabetes UK (charity), Juvenile Diabetes Research Foundation (charity), and the European Foundation for the Study of Diabetes (charity). BMS is also a member of the Diabetes UK research grant committee. Representatives from GSK, Takeda, Janssen, Quintiles, AstraZeneca, and Sanofi have attended meetings as part of the industry group involved with the MASTERMIND consortium. All declarations are outside of this study. LMG, ATH, KGY, PC and JMD have nothing to declare.

## Appendix A Supplementary data

Supplementary Material

MASTERMIND_Consortium_Members_PubMed
