# Supplementary material for: Validation of an algorithm for selection of SGLT2 and DPP4 inhibitor therapies in people with type 2 diabetes across major UK ethnicity groups: a retrospective cohort study
Source: Lancet Reg Health Eur. 2025 Nov 27;61:101547. doi: 10.1016/j.lanepe.2025.101547 (PMC12702077; doi:10.1016/j.lanepe.2025.101547)
Supplement: Supplementary file 1 — Supplementary Material [file mmc1.pdf]

Validation of an algorithm for selection of SGLT2 and DPP4 inhibitor therapies in people with type 2 diabetes across major UK ethnicity groups: a retrospective cohort study

Authors:

Laura M Güdemann (PhD), Katherine G Young (PhD), Pedro Cardoso (PhD), Bilal A Mateen (MBBS) (Prof), Rury R Holman (F.Med.Sci) (Prof), Naveed Sattar (MD) (Prof), Ewan R Pearson (PhD) (Prof), Andrew T Hattersley (DM) (Prof), Angus G Jones (PhD) (Prof), Beverley M Shields (PhD), John M Dennis (PhD), on behalf of the MASTERMIND consortium

Table of Contents

Technical summary of the prediction model Dennis et al. (2022) 3

Explanation of the testing procedure to assess the need for model adjustment 4

sFigure 1: Flowchart 5

sTable 1: Baseline clinical characteristics of the weight change outcome specific study cohort. 6

sTable 2: Baseline clinical characteristics of the discontinuation outcome specific study cohort. 7

sTable 3: Results of the model updating testing procedure. 8

sFigure 2: Assessment of calibration between model predicted HbA1c 6 month outcome and observed HbA1c. 9

sTable 4: Validation of 6-month HbA1c: Observed treatment effects across subgroups defined by clinical cut-offs of predicted treatment effects. 10

sFigure 3: Calibration of predicted 6-month HbA1c benefit, by UK Mixed and Other ethnicity group separately. 12

sFigure 4: Calibration of predicted 6-month HbA1c benefit, by UK ethnicity group controlling for deprivation deciles in the estimation of predicted HbA1c differences (mmol/mol). 13

sFigure 5: Calibration of predicted 12-month HbA1c benefit, by UK ethnicity group. 14

sTable 5: Estimates of 6-month weight change. 15

sTable 6: Estimates of treatment discontinuation within 6-months. 17

MASTERMIND consortium members 19

References 20

Technical summary of the prediction model Dennis et al. (2022)

Development data:
The treatment selection model for SGLT2i and DPP4i therapies in people with type 2 diabetes (T2D) was developed in UK primary care records from the Clinical Practice Research Datalink. Dennis et al. 2022 [1] identified new users (n = 5,374 SGLT2i; n = 8,695 DPP4i) of the study treatments. Individuals who initiation the study treatments as first line treatment or on the same day as insulin were excluded from the study. Additionally, individuals were excluded if they had no valid outcome HbA1c data recorded, had missing baseline HbA1c, baseline HbA1c < 53 mmol/mol / > 120 mmol/mol, or baseline eGFR < 45 mL/min/1.73m^2. A summary of the study cohort can be found in Dennis et al. (2022), supplementary material sFlowchart.[1]

Prediction model equation:

HbA1c outcome = β_0 + β_1(drugclass) + β_2(n therapies ever taken) + β_3(n current therapies) + β_4(baseline eGFR) + β_5(log(baseline ALT)) + β_6(baseline BMI) + β_7(age at treatment initiation) + f_1(baseline HbA1c) x drugclass + f_2(baseline eGFR) x drugclass + $f_3$log((ALT)) x drugclass + f_4(baseline BMI) x drugclass + f_5(age at treatment initiation) x drugclass + f_6(month of HbA1c) + ε

- β_0 is the intercept.
- $\beta_1, \ldots, \beta_7$ are the coefficients for the linear terms.
- $f_1, \ldots, f_6$ are functions representing 3-knot restricted cubic splines for the corresponding variables
- ε is the error term.

The spline terms which interact with the variable drugclass such as f_1(baseline HbA1c) indicate that the effect of these variables on the outcomes depend on the drug class. [1]

Predictor selection:

Associations of clinical features with differential HbA1c outcome on the two study treatments were sequentially identified starting with a linear regression of outcome HbA1c on a drug-by-baseline HbA1c interaction which was modelled as a 3-knot restricted cubic split in order to allow for non-linearity. Additionally, the model controlled for differences of drug order, number of concurrent and ever prescribed glucose-lowering treatments and month of outcome measurement (month outcome HbA1c was recorded relative to baseline HbA1c measurement). On the base of this first model, associations of other baseline clinical features with differential HbA1c response was assessed by adding each feature in turn with a drug-by-baseline characteristic interaction term. The final treatment selection model was identified via model evaluation (examination of normality of residuals and linearity of associations for continuous variables) and after a series of sensitivity analyses to evaluate consistency of associations. Model validation was executed on a 40% hold out sample of the CPRD cohort and in data of 14 clinical trials.[1]

Explanation of the testing procedure to assess the need for model adjustment

The procedure employed in this study is informed by Vergouwe et al. (2017) [2] and relies on log-likelihood tests sequentially comparing the three fitted models as described below. The R code for this testing procedure can be found online at: https://github.com/Exeter-Diabetes/CPRD-LauraJohn-SGLT2iDPPi-ethnicity. Below the testing procedure is summarized with an example model including two continuous predictor variables (X_1, X_2) of the outcome (Y) and no interaction terms, for simplicity.

Model 1: Original model

$$Y = \beta_{0,\, original\, model} + \beta_{1,\, original\, model} * X_1 + \beta_{2,\, original\, model} * X_2$$

Model 2: Recalibration-in-the-large

Only the intercept of the original model is updated for this model as a free parameter. This will adjust the average outcome level. **X** in this model represents the linear predictor based on the original model which is included as an offset in this model (i.e. the slope is fixed at unity) [2].

$$Y = \beta_{0,\, new} + \boldsymbol{X}$$

Model 3: Intercept/slope recalibration

This model includes updating the intercept and the overall calibration slope.

$$Y = \beta_{0,new} + \beta_{new,\, overall} * \boldsymbol{X}$$

Sequential testing procedure is based on the following model comparisons and decision rules.

Model comparisons:

	**Models compared**	**Degrees of freedom**
**Test 1**	model 1 vs model 2	m
**Test 2**	model 2 vs model 3	m + 1

Where m is the number of estimated parameters in the original model (intercept not considered). [2] For models including categorical variables, this number might differ for the same model but different data subsets (e.g. ethnicity x drug class subsets) as it depends on the coverage of variable categories in the study data.

Decision rules:

**Test 1**	**Test 2**	**Model choice**
not significant	not significant	model 1
significant	not significant	model 2
not significant	significant	model 3

sFigure 1: Flowchart

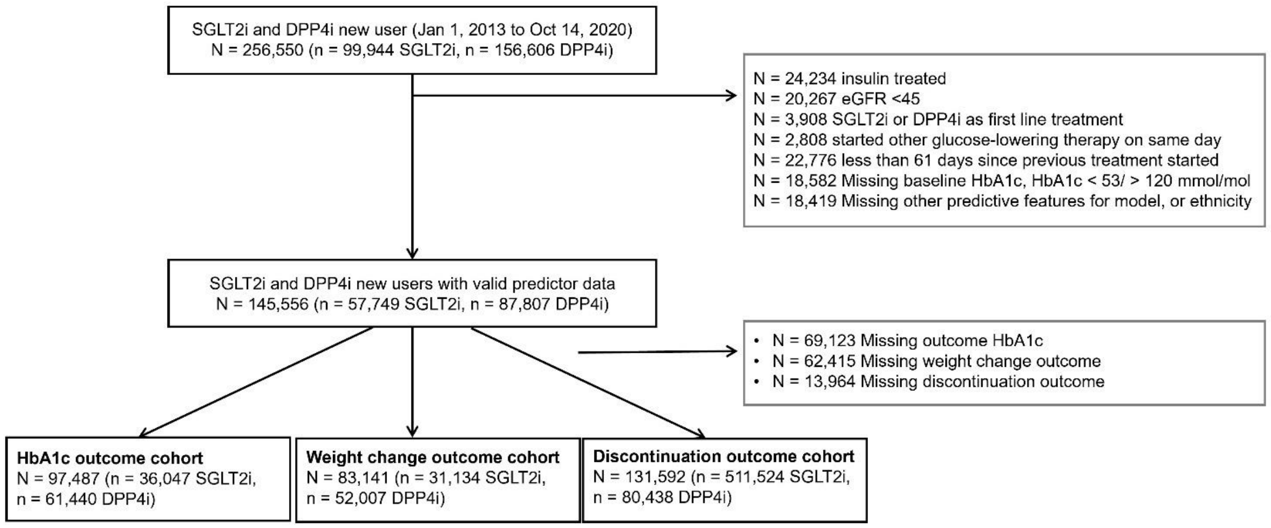

sTable 1: Baseline clinical characteristics of the weight change outcome specific study cohort. Data are mean (1 SD) for continuous variables.

	**DPP4i (N = 52,007)**	**SGLT2i (N = 31,134)**
**Current age, years**	62.7 (11.9)	58.4 (10.3)
**Duration of diabetes, years**	8.8 (6.6)	9.1 (6.3)
**Sex**		
Female	19,966 (38.5%)	11,677 (37.5%)
Male	32,041 (61.6%)	19,457 (62.5%)
**Ethnicity**		
White	41,676 (80.1%)	24,846 (79.8%)
South Asian	6,878 (13.2%)	4,326 (13.9%)
Black	2,267 (4.4%)	1,192 (3.8%)
Mixed or other	1,186 (2.3%)	770 (2.5%)
**Index of multiple deprivation quintile**		
1 (least deprived)	8,759 (16.8%)	5,292 (17.0%)
2	9,440 (18.2%)	5,551 (17.8%)
3	9,927 (19.1%)	6,048 (19.4%)
4	11,586 (22.3%)	6,866 (22.1%)
5 (most deprived)	12,267 (23.6%)	7,361 (23.6%)
Not available	28 (0.05%)	16 (0.05%)
**DPP4-inhibitor type**		
Alogliptin	10,960 (21.1%)	-
Linagliptin	10,811 (20.8%)	-
Saxagliptin	3,231 (6.2%)	-
Sitagliptin	26,554 (51.1%)	-
Vildagliptin	451 (0.9%)	-
**SGLT2-inhibitor type**		
Canagliflozin	-	5,644 (18.1%)
Dapagliflozin	-	13,632 (43.8%)
Empagliflozin	-	11,837 (38.0%)
Ertugliflozin	-	21 (0.1%)
**Number of glucose-lowering drug classes ever prescribed**		
2	23,482 (45.2%)	7,422 (23.8%)
3	21,980 (42.3%)	9,791 (31.4%)
4+	6,545 (12.6%)	13,921 (44.7%)
**Number of other current glucose-lowering drugs**		
0		
1	2,772 (5.3%)	994 (3.2%)
2	30,594 (58.8%)	12,606 (40.5%)
3	18,046 (34.7%)	14,078 (45.2%)
	595 (1.1%)	3,456 (11.1%)
**Background therapy**		
Metformin	46,870 (90.1%)	28,576 (91.8%)
Sulfonylurea	18,838 (36.2%)	11,123 (35.7%)
DPP4-inhibitor	-	9,005 (28.9%)
SGLT2-inhibitor	1,409 (2.7%)	-
Thiazolidinedione	1,075 (2.1%)	776 (2.5%)
GLP-1 receptor agonist	167 (0.3%)	1,644 (5.3%)
**Baseline biomarkers**		
HbA1c mmol/mol	71.9 (13.3)	75.8 (14.3)
BMI, kg/m^2	31.9 (6.4)	33.8 (6.7)
eGFR, mL/min per 1.73 m^2	88.7 (17.7)	95.2 (14.5)
Alanine transaminase, IU/L	32.3 (19.3)	34.9 (20.1)
**Weight outcome**		
Achieved weight (kg)	90.4 (20.4)	93.8 (20.9)

sTable 2: Baseline clinical characteristics of the discontinuation outcome specific study cohort. Data are mean (1 SD) for continuous variables.

	DPP4i (N = 80,438)	SGLT2i (N = 51,124)
**Current age, years**	62.6 (12.1)	58.5 (10.5)
**Duration of diabetes, years**	8.91 (6.69)	9.29 (6.30)
**Sex**		
Female	31,617 (39.3%)	19,845 (38.8%)
Male	48,821 (60.7%)	31,309 (61.2%)
**Ethnicity**		
White	63,498 (78.9%)	40,254 (78.7%)
South Asian	11,242 (14.0%)	7,511 (14.7%)
Black	3,775 (4.7%)	2,069 (4.0%)
Mixed or other	1,923 (2.4%)	1,320 (2.6%)
**Index of multiple deprivation quintile**		
1 (least deprived)	13,301 (16.5%)	8,544 (16.7%)
2	14,471 (18.0%)	9,100 (17.8%)
3	15,358 (19.1%)	9,856 (19.3%)
4	18,057 (22.4%)	11,456 (22.4%)
5 (most deprived)	19,204 (23.9%)	12,168 (23.8%)
Not available	47 (0.06%)	0 (0%)
**DPP4-inhibitor type**		
Alogliptin	16,922 (21.0%)	-
Linagliptin	17,042 (21.2%)	-
Saxagliptin	4,757 (5.9%)	-
Sitagliptin	41,054 (51.0%)	-
Vildagliptin	663 (0.8%)	-
**SGLT2-inhibitor type**		
Canagliflozin	-	
Dapagliflozin	-	
Empagliflozin	-	
Ertugliflozin	-	
**Number of glucose-lowering drug classes ever prescribed**		
2	35,809 (44.5%)	11,968 (23.4%)
3	34,040 (42.3%)	16,014 (31.3%)
4+	10,589 (13.2%)	23,172 (45.3%)
**Number of other current glucose-lowering drugs**		
0		
1	5,092 (6.3%)	1,991 (3.9%)
2	47,043 (58.5%)	20,289 (39.7%)
3	27,191 (33.8%)	22,286 (43.6%)
	1,112 (1.4%)	6,588 (12.9%)
**Background therapy**		
Metformin	71,273 (88.6%)	46,134 (90.2%)
Sulfonylurea	28,812 (35.8%)	18,824 (36.8%)
DPP4-inhibitor	-	15,492 (30.3%)
SGLT2-inhibitor	2,341 (2.9%)	-
Thiazolidinedione	1,761 (2.2%)	1,441 (2.8%)
GLP-1 receptor agonist	380 (0.5%)	2,766 (5.4%)
**Baseline biomarkers**		
HbA1c mmol/mol	72.6 (13.8)	76.2 (14.6)
BMI, kg/m^2	31.9 (6.51)	33.6 (6.76)
eGFR, mL/min per 1.3 m^2	88.8 (17.9)	95.0 (14.8)
Alanine transaminase, IU/L	32.3 (19.3)	34.9 (20.1)
**Discontinuation outcome**		
Discontinuation, yes	13,356 (16.6%)	10,026 (19.6%)

sTable 3: Results of the model updating testing procedure.

Cohort		N	P-values		Final model chosen	log-likelihood	Updated intercept (95% CI)*
Treatment	Ethnicity		Test 1	Test 2			
DPP4-inhibitors	White	48,832	< 0.0001	0.14	Updated intercept	-194,208	-1.6 (-1.7, -1.5)
SGLT2-inhibitors	White	28,497	< 0.0001	0.38	Updated intercept	-110,538	-0.9 (-1.0, -0.7)
DPP4-inhibitors	Black	2,780	< 0.0001	0.79	Updated intercept	-11,644	-3.0 (-3.6, -2.4)
SGLT2-inhibitors	Black	1,409	< 0.0001	1	Original	-5,765	NA
DPP4-inhibitors	South Asian	8,397	1	1	Updated intercept	-33,076	-2.6 (-2.8, -2.3)
SGLT2-inhibitors	South Asian	5,209	1	1	Original	-20,360	NA
DPP4-inhibitors	Other	906	0.05	1	Original	-3,626	NA
SGLT2-inhibitors	Mixed/Other	932	1	1	Original	-3,625	NA

Test 1 compared the prediction performance of Model 1 (original model) versus Model 2 (recalibration-in-the-large model). Test 2 compared Model 2 with Model 3 (updated intercept and slope). If the second test was significant, Model 3 was chosen, if the first but not the second test was significant, Model 2 was chosen, and if none of the tests were significant, Model 1 was chosen.

*A negative value represents greater response than predicted by the original model

sFigure 2: Assessment of calibration between model predicted HbA1c 6 month outcome and observed HbA1c. Green dotted line: perfect calibration, yellow line: observed calibration.

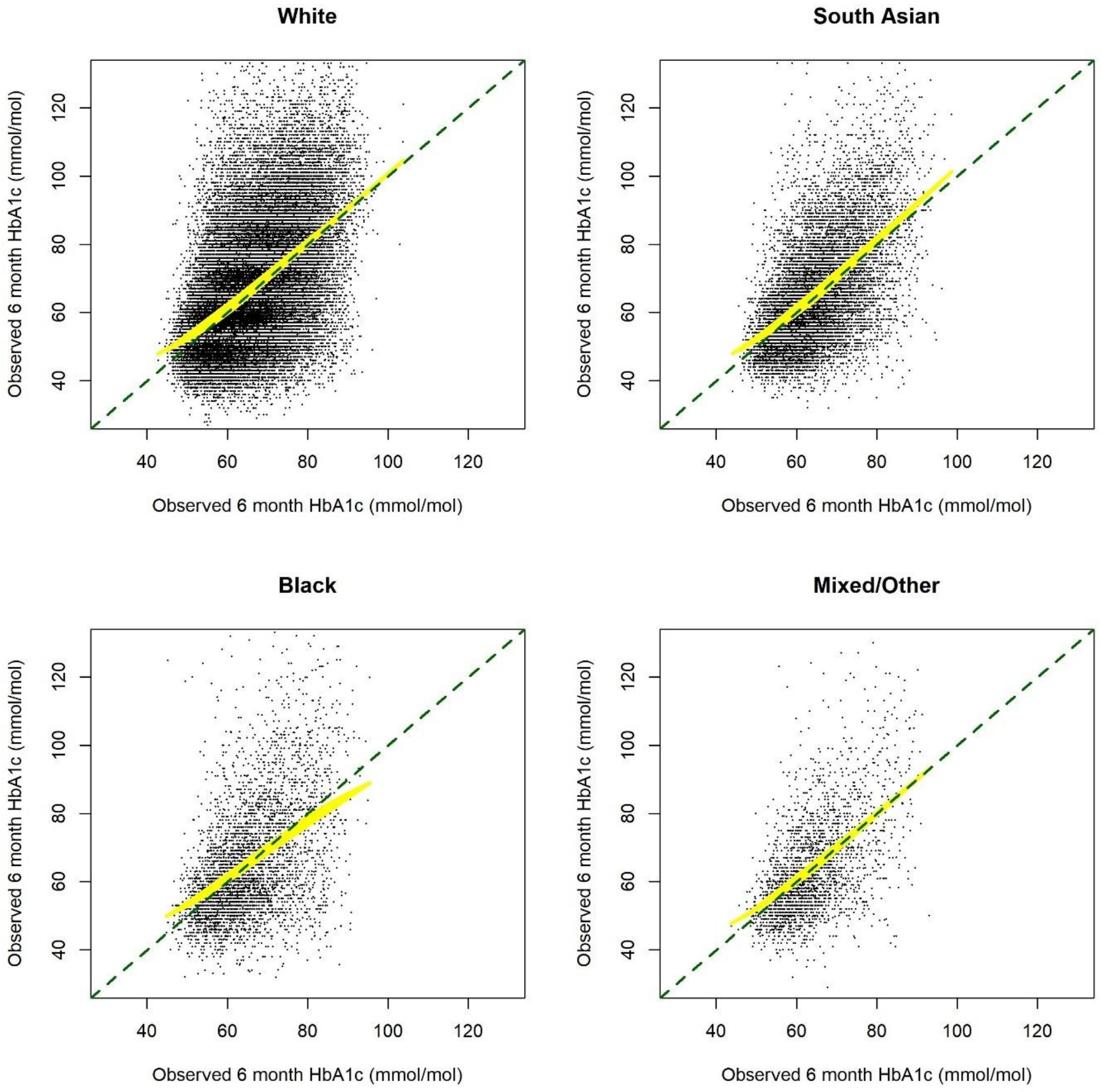

sTable 4: Validation of 6-month HbA1c: Observed treatment effects across subgroups defined by clinical cut-offs of predicted treatment effects. Estimates include all patients with valid baseline data for glucose-lowering treatment selection model and with glycaemic benefit outcome recorded between 3 and 15 months after drug initiation, on unchanged glucose-lowering therapy. Estimates are unadjusted.

			**Achieved HbA1c difference (mmol/mol)**	
**Ethnicity**	**predicted HbA1c difference**	**N patients**	**DPP4i**	**SGLT2i**
**White**	SGLT2i benefit by ≥5 mmol/mol	26,940	-10.6 (-10.8; -10.3)	-16.8 (-17.0; -16.5)
	SGLT2i benefit by 3-5 mmol/mol	15,840	-8.5 (-8.8; -8.2)	-10.7 (-11.0; -10.4)
	SGLT2i benefit by 0-3 mmol/mol	18,998	-7.7 (-7.9; -7.5)	-6.7 (-7.0; -6.4)
	DPP4i benefit by 0-3 mmol/mol	10,431	-7.1 (-7.4; -6.9)	-3.7 (-4.2; -3.2)
	DPP4i benefit by ≥3 mmol/mol	5,120	-5.4 (-5.8; -5.1)	-0.7 (-1.9; 0.5)
**South Asian**	SGLT2i benefit by ≥5 mmol/mol	2,498	-11.4 (-12.3; -10.6)	-17.7 (-18.6; -16.9)
	SGLT2i benefit by 3-5 mmol/mol	3,051	-10.3 (-11.0; -9.6)	-12.9 (-13.6; -12.2)
	SGLT2i benefit by 0-3 mmol/mol	4,395	-8.3 (-8.7; -7.8)	-8.0 (-8.6; -7.4)
	DPP4i benefit by 0-3 mmol/mol	2,510	-7.1 (-7.6; -6.5)	-4.8 (-5.7; -4.0)
	DPP4i benefit by ≥3 mmol/mol	1,152	-6.5 (-7.2; -5.8)	-1.5 (-3.0; 0.0)
**Black**	SGLT2i benefit by ≥5 mmol/mol	348	-15.5 (-18.3; -12.7)	-18.6 (-21.4; -15.8)
	SGLT2i benefit by 3-5 mmol/mol	823	-12.3 (-14.0; -10.5)	-16.9 (-18.7; -15.0)
	SGLT2i benefit by 0-3 mmol/mol	1,473	-11.5 (-12.6; -10.3)	-9.9 (-11.3; -8.4)
	DPP4i benefit by 0-3 mmol/mol	982	-8.5 (-9.6; -7.3)	-5.4 (-6.9; -4.0)
	DPP4i benefit by ≥3 mmol/mol	563	-7.6 (-8.9; -6.2)	-3.4 (-7.0; 0.2)
**Mixed/Other**	SGLT2i benefit by ≥5 mmol/mol	423	-10.2 (-12.6; -7.8)	-17.1 (-19.1; -15.1)
	SGLT2i benefit by 3-5 mmol/mol	508	-11.1 (-13.0; -9.2)	-14.5 (-16.2; -12.8)
	SGLT2i benefit by 0-3 mmol/mol	749	-9.8 (-11.1; -8.6)	-9.6 (-11.0; -8.1)
	DPP4i benefit by 0-3 mmol/mol	458	-7.1 (-8.4; -5.8)	-5.7 (-7.7; -3.7)

	DPP4i benefit by ≥3 mmol/mol	225	-6.7 (-8.6; -4.8)	-0.8 (-4.4; 2.6)

sFigure 3: Calibration of predicted 6-month HbA1c benefit, by UK Mixed and Other ethnicity group separately. Red lines represent perfect calibration. Point estimates represent treatment effects for subgroups defined by decile of predicted treatment benefit. Bars represent 95% confidence intervals. Negative values reflect a predicted 6-month HbA1c benefit on SGLT2-inhibitor treatment, positive values reflect a predicted 6-month HbA1c on DPP4-inhibitor treatment.[1]

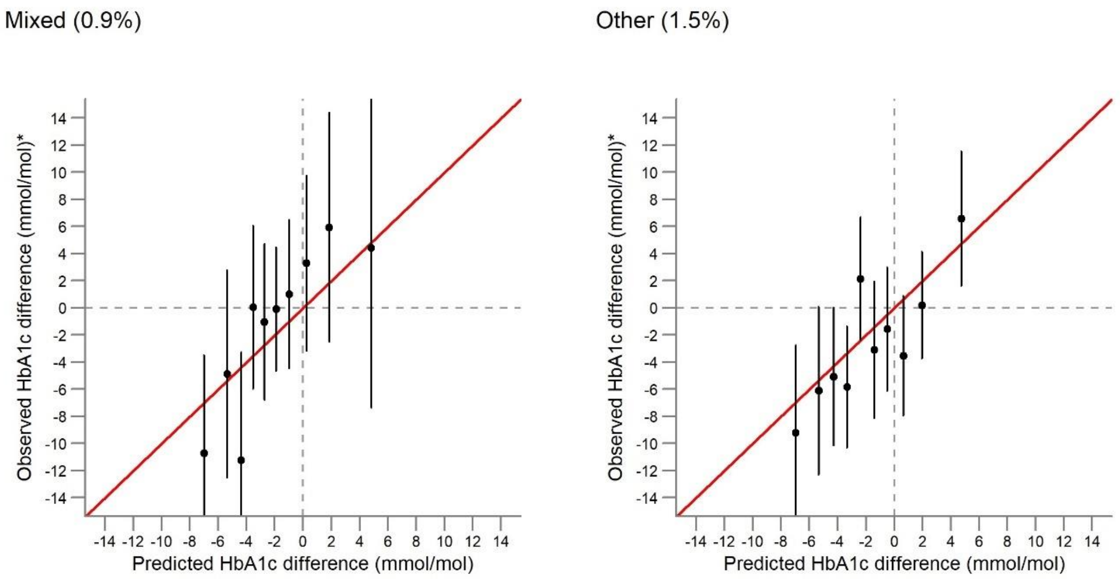

[1] Average HbA1c differences are adjusted absolute mean differences in 6-month HbA1c outcome between individuals receiving each drug class.

sFigure 4: Calibration of predicted 6-month HbA1c benefit, by UK ethnicity group controlling for deprivation deciles in the estimation of predicted HbA1c differences (mmol/mol). N SGL2i = 36,030; N DPP4i = 61,367, representing 99.9% of the overall study cohort for HbA1c outcome. Red lines represent perfect calibration. Point estimates represent treatment effects for subgroups defined by decile of predicted treatment benefit. Bars represent 95% confidence intervals. Negative values reflect a predicted 6-month HbA1c benefit on SGLT2-inhibitor treatment, positive values reflect a predicted 6-month HbA1c on DPP4-inhibitor treatment.[2]

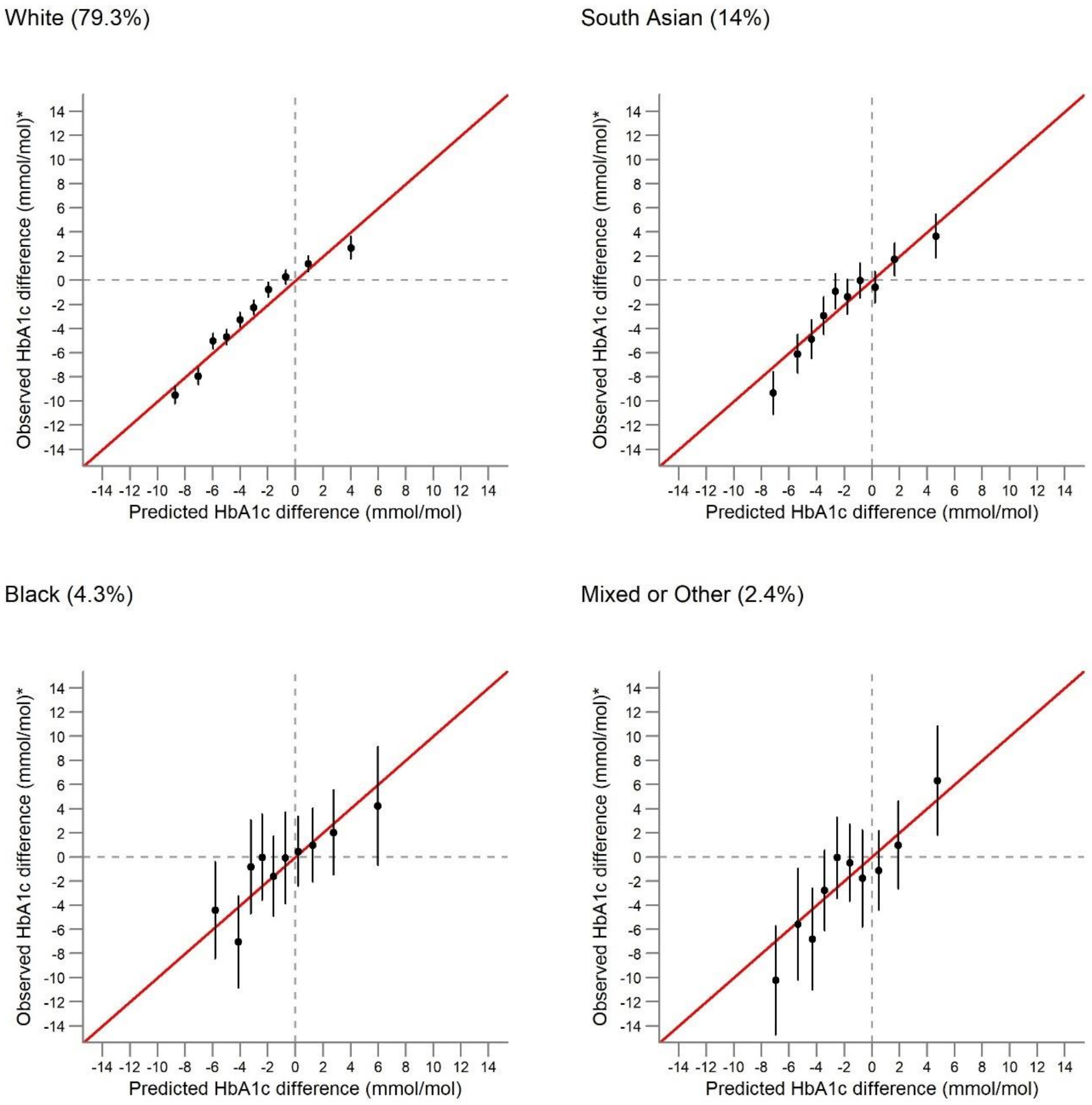

[2] Average HbA1c differences are adjusted absolute mean differences in 6-month HbA1c outcome between individuals receiving each drug class.

sFigure 5: Calibration of predicted 12-month HbA1c benefit, by UK ethnicity group. Red lines represent perfect calibration. Point estimates represent treatment effects for subgroups defined by decile of predicted treatment benefit. Bars represent 95% confidence intervals. Negative values reflect a predicted 12-month HbA1c benefit on SGLT2-inhibitor treatment, positive values reflect a predicted 12-month HbA1c on DPP4-inhibitor treatment.[3]

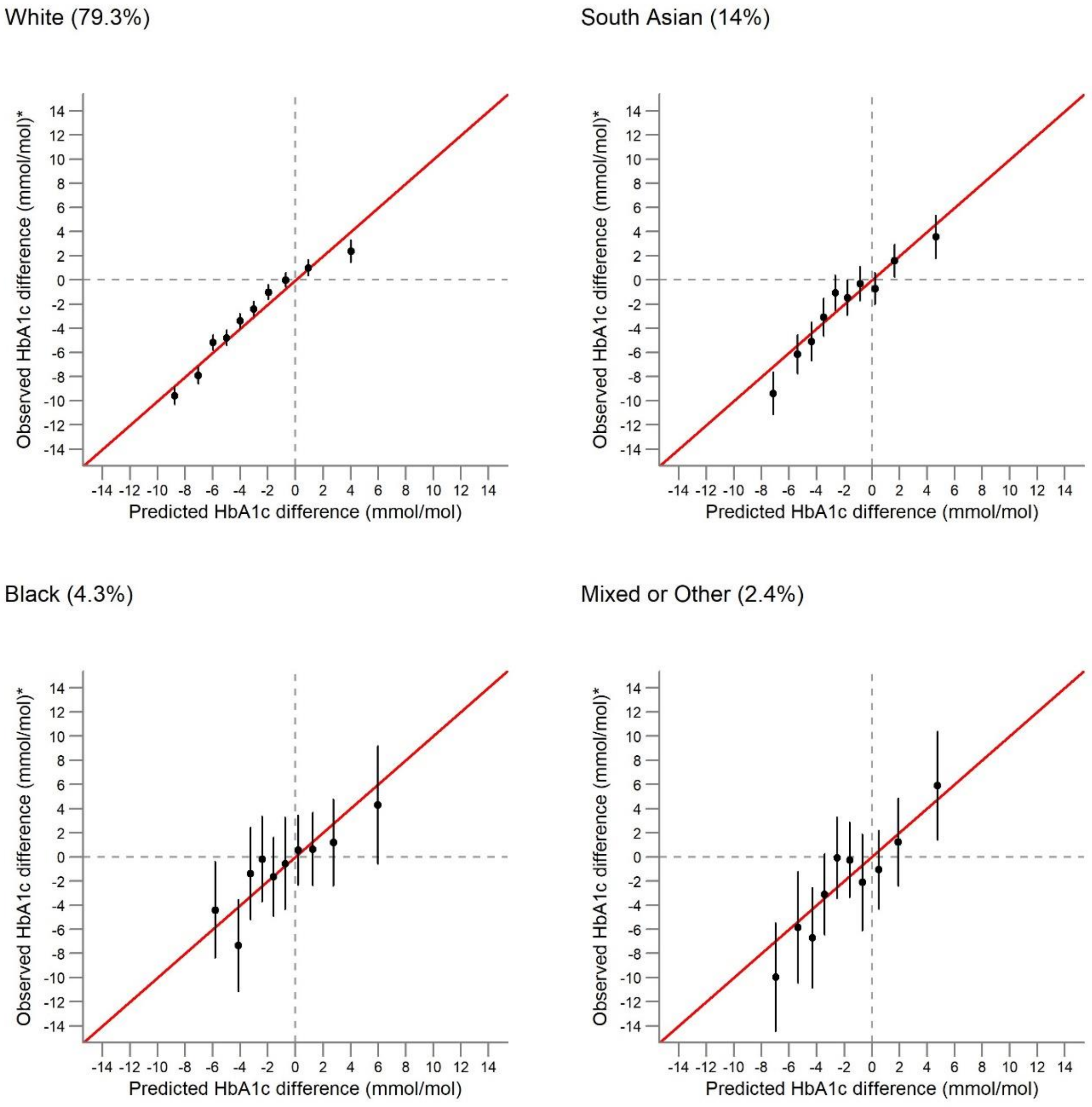

[3] Average HbA1c differences are adjusted absolute mean differences in 6-month HbA1c outcome between individuals receiving each drug class.

sTable 5: Estimates of 6-month weight change. Estimates include all patients with valid baseline data for glucose-lowering treatment selection model and with weight outcome recorded between 3 and 15 months after drug initiation, on unchanged glucose-lowering therapy. Estimates are adjusted for baseline weight, the number of currently prescribed glucose-lowering treatments, and the number of glucose-lowering drug classes ever prescribed.

			**Weight change in kg (Median [IQR])**	
**Ethnicity**	**predicted HbA1c difference**	**N patients**	**DPP4i**	**SGLT2i**
**White**	SGLT2i benefit by ≥5 mmol/mol	23,246	-1.2 (-1.3; -1.1)	-3.9 (-4.0; -3.9)
	SGLT2i benefit by 3-5 mmol/mol	13,667	-1.0 (-1.1; -0.9)	-4.2 (-4.3; -4.1)
	SGLT2i benefit by 0-3 mmol/mol	16,469	-0.9 (-1.0; -0.9)	-4.2 (-4.3; -4.0)
	DPP4i benefit by 0-3 mmol/mol	8,877	-1.1 (-1.2; -1.0)	-3.9 (-4.1; -3.6)
	DPP4i benefit by ≥3 mmol/mol	4,263	-1.0 (-1.2; -0.9)	-3.5 (-4.0; -3.1)
**South Asian**	SGLT2i benefit by ≥5 mmol/mol	2,089	-0.9 (-1.1; -0.7)	-3.2 (-3.5; -2.9)
	SGLT2i benefit by 3-5 mmol/mol	2,527	-0.9 (-1.1; -0.6)	-3.1 (-3.3; -2.9)
	SGLT2i benefit by 0-3 mmol/mol	3,586	-0.6 (-0.8; -0.5)	-2.9 (-3.1; -2.7)
	DPP4i benefit by 0-3 mmol/mol	2,084	-0.8 (-1.0; -0.6)	-2.8 (-3.1; -2.4)
	DPP4i benefit by ≥3 mmol/mol	918	-0.9 (-1.1; -0.6)	-2.7 (-3.3; -2.0)
**Black**	SGLT2i benefit by ≥5 mmol/mol	309	-0.6 (-1.2; 0.0)	-3.0 (-3.8; -2.1)
	SGLT2i benefit by 3-5 mmol/mol	663	-0.1 (-0.6; 0.4)	-2.6 (-3.0; -2.2)
	SGLT2i benefit by 0-3 mmol/mol	1,214	0.0 (-0.3; 0.4)	-2.8 (-3.1; -2.4)
	DPP4i benefit by 0-3 mmol/mol	813	-0.8 (-1.6; -0.3)	-2.7 (-3.1; -2.2)
	DPP4i benefit by ≥3 mmol/mol	460	-0.5 (-1.0; 0.1)	-2.3 (-3.3; -1.3)
**Mixed/Other**	SGLT2i benefit by ≥5 mmol/mol	354	-1.2 (-2.0; -0.4)	-3.2 (-4.1; -2.4)
	SGLT2i benefit by 3-5 mmol/mol	430	-0.9 (-1.5; -0.4)	-2.4 (-3.1; -1.8)
	SGLT2i benefit by 0-3 mmol/mol	609	-0.7 (-1.1; -0.3)	-2.9 (-3.4; -2.5)
	DPP4i benefit by 0-3 mmol/mol	366	-0.6 (-1.0; -0.1)	-3.4 (-4.1; -2.7)

	DPP4i benefit by ≥3 mmol/mol	197	-1.1 (-1.6; -0.6)	-2.6 (-3.7; -1.4)

sTable 6: Estimates of treatment discontinuation within 6-months. Estimates include all patients with valid baseline data for glucose-lowering treatment selection model and with 3 additional months of follow up to confirm treatment was truly discontinued. Estimates are adjusted for the number of currently prescribed glucose-lowering treatments, and the number of glucose-lowering drug classes ever prescribed.

			**Treatment discontinuation % (Median [IQR])**	
**Ethnicity**	**predicted HbA1c difference**	**N patients**	**DPP4i**	**SGLT2i**
**White**	SGLT2i benefit by ≥5 mmol/mol	36,872	16.7 (16.2; 17.3)	16.2 (15.6; 16.7)
	SGLT2i benefit by 3-5 mmol/mol	21,133	15.9 (15.2; 16.5)	19.2 (18.4; 20.0)
	SGLT2i benefit by 0-3 mmol/mol	25,154	15.8 (15.3; 16.4)	22.5 (21.6; 23.3)
	DPP4i benefit by 0-3 mmol/mol	13,833	16.2 (15.5; 16.9)	25.8 (24.3; 27.3)
	DPP4i benefit by ≥3 mmol/mol	6,760	15.5 (14.6; 16.4)	34.4 (30.7; 38.2)
**South Asian**	SGLT2i benefit by ≥5 mmol/mol	3,477	19.3 (17.5; 21.2)	16.7 (15.0; 18.5)
	SGLT2i benefit by 3-5 mmol/mol	4,127	16.0 (14.5; 17.5)	18.4 (16.7; 20.2)
	SGLT2i benefit by 0-3 mmol/mol	6,011	18.3 (17.1; 19.6)	23.3 (21.7; 25.0)
	DPP4i benefit by 0-3 mmol/mol	3,495	19.5 (17.9; 21.1)	23.3 (21.0; 25.8)
	DPP4i benefit by ≥3 mmol/mol	1,643	18.4 (16.4; 20.6)	30.5 (25.6; 35.9)
**Black**	SGLT2i benefit by ≥5 mmol/mol	505	18.5 (14.4; 23.5)	19.2 (14.6; 24.9)
	SGLT2i benefit by 3-5 mmol/mol	1,147	19.6 (16.8; 22.8)	20.3 (16.9; 24.1)
	SGLT2i benefit by 0-3 mmol/mol	2,021	19.0 (16.9; 21.3)	21.2 (18.5; 24.2)
	DPP4i benefit by 0-3 mmol/mol	1,389	21.2 (15.0; 20.8)	24.6 (20.9; 28.7)
	DPP4i benefit by ≥3 mmol/mol	782	17.7 (15.0; 20.8)	28.7 (21.2; 37.5)
**Mixed/Other**	SGLT2i benefit by ≥5 mmol/mol	578	16.7 (12.9; 21.5)	14.1 (10.6; 18.6)
	SGLT2i benefit by 3-5 mmol/mol	719	19.7 (15.9; 24.2)	20.1 (16.3; 24.5)
	SGLT2i benefit by 0-3 mmol/mol	1,012	17.3 (14.5; 20.6)	19.7 (16.1; 23.8)
	DPP4i benefit by 0-3 mmol/mol	621	17.9 (14.5; 21.8)	21.6 (16.3; 28.0)

	DPP4i benefit by ≥3 mmol/mol	313	15.2 (11.3; 20.2)	27.0 (17.6; 39.0)

MASTERMIND consortium members

Prof Andrew Hattersley[1], Prof Ewan Pearson[2], Prof Angus Jones [1], Prof Beverley Shields [1], Prof John Dennis [1], Dr Lauren Rodgers [1], Prof William Henley [1], Prof Timothy McDonald [1], Prof Michael Weedon [1], Prof Nicky Britten [1], Catherine Angwin [1], Prof Naveed Sattar[3], Dr Robert Lindsay[3], Prof Christopher Jennison[4], Prof Mark Walker[5], Prof Kennedy Cruickshank[6], Dr Salim Janmohamed[7], Prof Christopher Hyde [1], Prof Rury Holman[8], Prof Andrew Farmer[8], Prof Alastair Gray[8], Prof Stephen Gough[8], Dr Olorunsola Agbaje[8], Dr Trevelyan McKinley [1], Dr Sebastian Vollmer[9], Dr Bilal Mateen[10], Prof William Hamilton [1], Dr Katherine Young [1], Rhian Hopkins [1], Dr Pedro Cardoso [1], Laura Güdemann [1]

[1] Clinical and Biomedical Sciences, University of Exeter Medical School, Exeter, UK

[2] Division of Diabetes, Endocrinology and Reproductive Medicine, Ninewells Hospital and Medical School, University of Dundee, Dundee, UK

[3] School of Cardiovascular and Metabolic Health, University of Glasgow, Glasgow, UK

[4] Department of Mathematical Sciences, University of Bath, Bath, UK

[5] Faculty of Medical Science, Medical School, University of Newcastle, Newcastle, UK

[6] Faculty of Life Sciences & Medicine, School of Cardiovascular and Metabolic Medicine & Sciences, Kings College London, London, UK

[7] GlaxoSmithKline, Heathrow, London, UK

[8] Radcliff Department of Medicine, University of Oxford, Oxford, UK

[9] Department of Computer Science, RPTU University Kaiserslautern-Landau , Kaiserslautern, Germany

[10] School of Life Sciences, University of Birmingham, Birmingham, UK

References

1. Dennis JM, Young KG, McGovern AP, Mateen BA, Vollmer SJ, Simpson MD, et al. Development of a treatment selection algorithm for SGLT2 and DPP-4 inhibitor therapies in people with type 2 diabetes: a retrospective cohort study. The Lancet Digital Health. 2022;4(12):e873-e83.

2. Vergouwe Y, Nieboer D, Oostenbrink R, Debray TP, Murray GD, Kattan MW, et al. A closed testing procedure to select an appropriate method for updating prediction models. Statistics in medicine. 2017;36(28):4529-39.
