# Supplementary material for: Validation of an algorithm for selection of SGLT2 and DPP4 inhibitor therapies in people with type 2 diabetes across major UK ethnicity groups: a retrospective cohort study
Source: Lancet Reg Health Eur. 2025 Nov 27;61:101547. doi: 10.1016/j.lanepe.2025.101547 (PMC12702077; doi:10.1016/j.lanepe.2025.101547)
Supplement: Supplementary file 2 — MASTERMIND_Consortium_Members_PubMed [file mmc2.docx]

## MASTERMIND consortium members

| **First and middle names** | **Surname** |
| --- | --- |
| Lauren | Rodgers |
| William | Henley |
| Timothy J. | McDonald |
| Michael N. | Weedon |
| Nicky | Britten |
| Catherine | Angwin |
| Robert S. | Lindsay |
| Christopher | Jennison |
| Mark | Walker |
| Kennedy | Cruickshank |
| Salim | Janmohamed |
| Christopher J. | Hyde |
| Andrew | Farmer |
| Alastair | Gray |
| Stephen | Gough |
| Olorunsola | Agbaje |
| Trevelyan J. | McKinley |
| Sebastian J. | Vollmer |
| William | Hamilton |
| Rhian | Hopkins |
